# Inhibition of ASIC1a Attenuates Neuronal Pyroptosis and Neuroinflammation Following Traumatic Brain Injury

**DOI:** 10.1002/cns.70918

**Published:** 2026-05-15

**Authors:** Jiawei Liang, Peng Chen, Yangyang Zhao, Pan Lei, Yulong Li, Yichen Zhang, Jia‐hong Cai, Yong‐an Jiang, Yan Zhang, Shiqi Cheng

**Affiliations:** ^1^ Department of Neurosurgery, The Second Affiliated Hospital, Jiangxi Medical College Nanchang University Nanchang Jiangxi China; ^2^ Department of Critical Care Medicine Nanjing Drum Tower Hospital Nanjing Jiangsu China; ^3^ Department of Neurosurgery, Jiangxi Provincial People's Hospital The First Affiliated Hospital of Nanchang Medical College Nanchang Jiangxi China; ^4^ Department of Plastic Surgery The Second Affiliated Hospital of Nanchang University Nanchang Jiangxi China; ^5^ Department of Rehabilitation Medicine, Jiangxi Provincial People's Hospital The First Affiliated Hospital of Nanchang Medical College Nanchang Jiangxi China; ^6^ Jiangxi Academy of Medical Science Nanchang Jiangxi China

**Keywords:** ASIC1a, NF‐κB, NLRP3, pyroptosis, traumatic brain injury

## Abstract

**Background:**

Traumatic brain injury (TBI) is a neurological disorder that severely affects health and function. Acid‐sensing ion channels 1a (ASIC1a), a proton‐gated cation channel permeable to Na^+^ and Ca^2+^, has been implicated in chronic neurodegeneration after TBI. However, its specific role in post‐TBI neuroinflammation remains poorly defined. In this study, we investigated the mechanistic involvement of ASIC1a in neuronal pyroptosis following TBI.

**Methods:**

Adult male C57BL/6 mice were subjected to controlled cortical impact (CCI) modeling using weight‐drop models. Expression of ASIC1a was evaluated by Western blot and immunofluorescence. Brain water content and neurobehavioral function were tested to assess brain edema and neurological performance. qRT‐PCR and Western blot were performed to evaluate the expression of pyroptosis and NF‐κB/NLRP3 pathway related markers. ELISA kits were used to measure the levels of IL‐1β and IL‐18.

**Results:**

Our results demonstrated that ASIC1a expression was upregulated following TBI and localized primarily in neurons. Furthermore, pharmacological inhibition of ASIC1a with PcTx1 significantly attenuated brain edema and improved neurological outcomes in TBI mice. Notably, inhibition of ASIC1a decreased the levels of the pyroptosis‐related protein GSDMD‐N, as well as the inflammatory cytokines IL‐1β and IL‐18. Importantly, ASIC1a blockade was associated with the downregulation of the NF‐κB/NLRP3 signaling pathway, suggesting that ASIC1a inhibition alleviates neuroinflammatory injury potentially through the modulation of this axis both in vivo and in vitro.

**Conclusion:**

Our findings implicate ASIC1a as an important modulator of neuroinflammatory progression following TBI. Furthermore, pharmacological inhibition of ASIC1a mitigates neuronal pyroptosis, an effect that is closely associated with the downregulation of the NF‐κB/NLRP3 axis.

## Graphical abstract

This study identifies ASIC1a as a key modulator of neuroinflammation following traumatic brain injury. Pharmacological inhibition of ASIC1a mitigates neuronal pyroptosis and improves neurological outcomes, a protective effect closely associated with the downregulation of the NF‐κB/NLRP3 signaling axis.

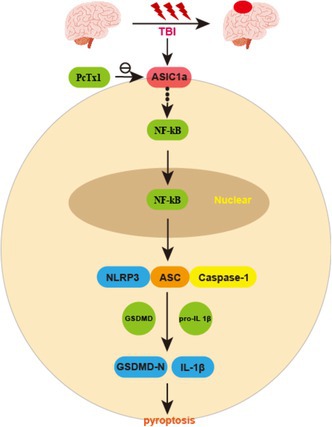

## 1 Introduction

Traumatic brain injury (TBI) constitutes an acute neurological condition characterized by significant physical and functional impairments, representing a leading global contributor to mortality and long‐term disability [1]. The pathophysiology of TBI involves biphasic injury mechanisms: primary injury resulting directly from mechanical forces during trauma, and secondary injury evolving over hours to days through cascading processes including neuroinflammation, cerebral edema, programmed cell death, and systemic inflammatory responses [2, 3, 4]. Although the precise molecular interplay underlying TBI pathogenesis remains incompletely elucidated, accumulating evidence positions dysregulated neuroinflammatory pathways as central mediators of secondary injury progression and functional outcomes [5, 6, 7].

Acid‐sensing ion channels (ASICs) constitute a family of proton‐gated sodium channels activated by extracellular acidosis in pathophysiological microenvironments [8, 9, 10]. So far, four distinct genes (ASIC1, ASIC2, ASIC3, and ASIC4) have been identified to encode seven functional subunits (ASIC1a, ASIC1b1, ASIC1b2, ASIC2a, ASIC2b, ASIC3, and ASIC4), which exhibit differential expression patterns in mammalian central and peripheral nervous systems [11, 12, 13]. Notably, ASIC1a predominantly localizes to central neurons, primarily assembling into heteromeric complexes such as ASIC1a homomers, ASIC1a/2a, and ASIC1a/2b isoforms [14, 15]. Accumulating evidence demonstrates that ASIC1a inhibition confers neuroprotection in ischemic brain injury models, significantly reducing infarct volume and neurological deficits [16, 17]. Meanwhile, growing evidence further indicates that ASIC1a plays an important role in neuroinflammation in degenerative diseases including AD, PD, and MS [18, 19]. Consistently, ASIC1a genetic knockout models exhibit attenuated chronic neuropathology and improved functional recovery post‐brain injury [20]. Despite these advances, the specific mechanistic involvement of ASIC1a in post‐TBI neuroinflammation—particularly its crosstalk with pyroptotic signaling pathways—remains poorly characterized, necessitating systematic investigation.

Inflammasome‐mediated pyroptosis, a form of cell‐programmed death, has emerged as a critical contributor to neuroinflammation after TBI [21, 22]. This process is characterized by cellular swelling, the formation of pores in the plasma membrane, and subsequent membrane rupture, leading to massive release of inflammatory cytokines and a pronounced inflammatory response [21, 23, 24]. The NLRP3 inflammasome, a multiprotein complex composed of NLRP3, apoptosis‐associated speck‐like protein containing a CARD (ASC), and pro‐caspase‐1, is widely distributed in both immune and non‐immune cells and serves as a master regulator of inflammatory responses [25, 26, 27]. Mechanistically, inflammasome activation triggers caspase‐1‐dependent cleavage of gasdermin D (GSDMD), generating N‐terminal fragments (GSDMD‐N) that form plasma membrane pores, thereby executing pyroptosis while facilitating mature IL‐1β/IL‐18 secretion [28, 29]. Nuclear factor‐κB (NF‐κB), a pleiotropic transcription factor, governs this process by transcriptionally upregulating both NLRP3 and pro‐IL‐1β during the priming phase [30, 31]. Recent evidence demonstrates that hydrogen‐rich saline attenuated subarachnoid hemorrhage‐induced early brain injury by inhibiting the NF‐κB/NLRP3 pathway [32]. Meanwhile, ASIC1a activation was shown to exacerbate renal ischemia–reperfusion injury through this same pathway [33]. However, it remains unclear whether ASIC1a potentiates post‐TBI neuroinflammation via NF‐κB/NLRP3‐mediated pyroptosis.

In this study, we investigated the relationship between ASIC1a and neuroinflammation following traumatic brain injury (TBI) using both in vivo and in vitro models. Our findings indicate that ASIC1a modulates TBI‐induced pyroptosis, an effect that is closely associated with the activation of the NF‐κB/NLRP3 signaling pathway. These results collectively suggest that pharmacological inhibition of ASIC1a holds promise as a potential therapeutic strategy for TBI treatment.

## 2 Materials and Methods

### 2.1 Animals

Adult male C57BL/6 mice, 6–8 weeks old, were purchased from GemPharmatech Co. Ltd. (Jiangsu, China) and housed in a controlled environment under a 12‐h light/dark cycle, regulated humidity, and temperature, and allowed free access to food and water. All animal procedures were approved by the Ethics Committee of the Second Affiliated Hospital of Nanchang University (Approval No. NCULAE‐20221108001, Nanchang University) and complied with the guidelines of the National Institute of Health.

### 2.2 Experimental Design

To characterize the temporal expression profile of ASIC1a post‐TBI, 30 C57BL/6 mice were randomly allocated into five experimental groups (*n* = 6 each group): sham, 12 h after TBI, 24 h after TBI, 3 days after TBI, and 5 days after TBI. Quantitative analysis of ASIC1a protein levels in peri‐contusional tissues was performed by western blotting. Spatial distribution was assessed through dual immunofluorescence staining at the 24 h timepoint (*n* = 3), examining co‐localization patterns between ASIC1a and cell‐specific markers: Iba‐1 (microglia), GFAP (astrocytes), and NeuN (neurons).

To evaluate the effect of ASIC1a after TBI, the ASIC1a‐specific inhibitor PcTx1 was delivered via intranasal administration 30 min pre‐TBI. To obtain the optimum dose of PcTx1, 30 mice were divided into five groups (*n* = 6 each group): sham, TBI, TBI + PcTx1 200 nM, TBI + PcTx1 400 nM, TBI + PcTx1 600 nM. Then, neurobehavioral function and brain water content were assessed at 24 h after TBI.

To determine whether ASIC1a amplifies pyroptosis and the potential mechanism of ASIC1a triggering pyroptosis after TBI, 54 mice were randomly divided into three groups: sham, TBI, and TBI + PcTx1. Western blot (*n* = 6 each group), ELISA (*n* = 6 each group), and PCR (*n* = 6 each group) were carried out at 24 h after TBI.

To determine whether ASIC1a triggers neuronal pyroptosis in vitro, HT‐22 cells were divided into three groups (*n* = 3 each group): controls, LPS‐stimulated, and LPS + PcTx1 pretreatment (20 nM, 1 h prior to 24 h coculture with LPS‐stimulated BV2 cells), followed by western blot analysis and ELISA.

**FIGURE 1:**
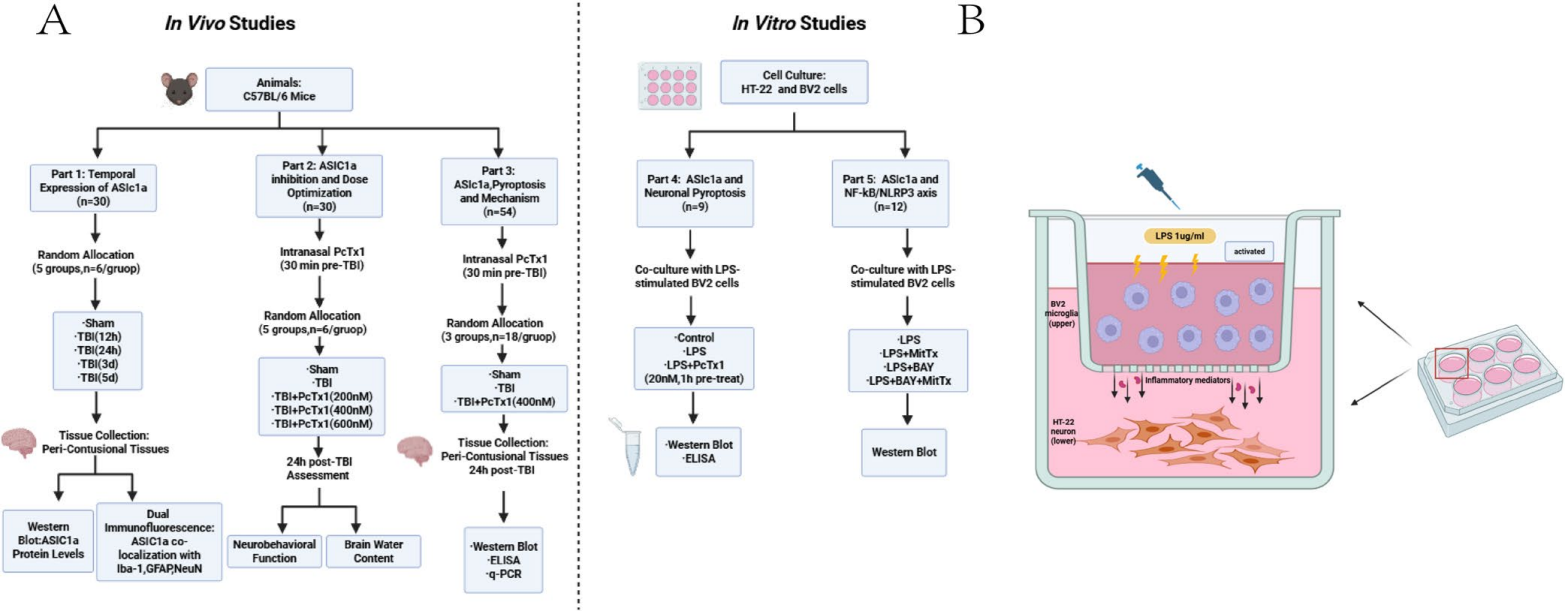
(A) A flowchart summarizing the overall study design. (B) An in vitro model of neuroinflammation.

To explore whether ASIC1a regulates NLRP3 inflammasome via the NF‐κB pathway in vitro, HT‐22 cells (coculture with LPS‐stimulated BV2 cells) were divided into four groups (*n* = 3 each group): LPS, LPS + MitTx, LPS + BAY 11‐7082, LPS + MitTx+BAY 11‐7082, followed by western blot analysis (Figure 1).

### 2.3 TBI Model

The controlled cortical impact (CCI) model was employed to induce traumatic brain injury. Mice were anesthetized with 3%–5% isoflurane (induction) followed by 1.5% maintenance through a nose cone while immobilized in a stereotaxic frame. A 4‐mm diameter craniotomy was performed at coordinates anteroposterior (AP) −2.0 mm and mediolateral (ML) +2.0 mm with careful preservation of the dura mater. Injury induction involved dropping a 20 g impactor from 25 cm height through a vertical guide tube, generating a controlled impact force of 250 g·cm onto the exposed dura. This produced a standardized cortical contusion (4 × 4 mm lesion) in the right parietal lobe, corresponding to moderate–severe TBI in the murine model. Post‐injury procedures included topical application of gentamicin sulfate (40,000 U, 4–5 drops), bone wax sealing of the craniotomy, and layered scalp closure. All surgical animals recovered on thermoregulated pads (37°C). Mice in the sham group received identical preparations excluding the CCI impact.

### 2.4 Drug Administration

PcTx1 (HY‐P1411, MedChemExpress), a selective ASIC1a inhibitor, was prepared in sterile normal saline solution. For in vivo administration, the compound was delivered via intranasal instillation 30 min prior to TBI induction. Based on a previous study [34], we systematically evaluated three concentrations (200 nM, 400 nM, and 600 nM) through the experiments of neurobehavioral function tests and brain water content measurement to determine the optimal therapeutic dose. In parallel in vitro studies, HT‐22 cells were pretreated with 20 nM PcTx1 (concentration established through CCK‐8 viability assays and a prior study [34]) for 1 h before commencing 24‐h coculture with LPS‐stimulated BV2 microglial cells.

### 2.5 Neurobehavioral Function Test

The modified Garcia score, forelimb placement test, and corner turn test were conducted at 24 h after TBI to evaluate neurological performance. The modified Garcia score (18‐point scale) evaluated six functional domains: (1) spontaneous activity, (2) tactile response, (3) limb movement symmetry, (4) proprioception, (5) forelimb extension, and (6) climbing ability. Each subtest was graded 0–3 (0 = absent, 3 = normal), with maximal scores indicating intact neurological function. The corner turn test was performed in a 30° angled apparatus, where mice completed 10 trials of free‐choice turning. Results were quantified as the percentage of right turns. The forelimb placement test was used to evaluate the response to vibrissae stimulation. The right forelimb was tested 10 times, expressed as a percentage of placing the left forelimb precisely on the edge of the test bench.

### 2.6 Brain Water Content

Cerebral edema was quantitatively assessed through gravimetric analysis at 24 h post‐TBI. Following euthanasia, brains were rapidly extracted and dissected into ipsilateral and contralateral hemispheric samples. Tissue samples were immediately weighed on a precision electronic balance (wet weight measurement) before being dehydrated at 100°C for 24 h in a temperature‐controlled oven. Post‐dehydration dry weights were obtained using the same calibrated balance. The percentage brain water content was calculated using the standard formula:

$$\left[\left(\mathrm{wet}\;\text{weight}-\mathrm{dry}\;\text{weight}\right)/\mathrm{wet}\;\text{weight}\right]\times 100\%.$$

### 2.7 Immunofluorescence Staining

TBI mice were deeply anesthetized and transcardially perfused with 0.9% NaCl followed by 4% paraformaldehyde (PFA). Coronal brain sections (10 μm thick) were prepared using a cryostat (RWD, China). Sections were blocked with QuickBlock Blocking Buffer for Immunol Staining (Beyotime, P0260, China) at room temperature for 10 min, and incubated overnight at 4°C with primary antibodies: ASIC1a (1:100; Proteintech, 27235‐1‐AP), NeuN (1:100, Proteintech, 26975‐1‐AP), Iba1 (1:200, Proteintech, 10904‐1‐AP), GFAP (1:200, Proteintech, 16825‐1‐AP), GSDMD‐N (1:100; Affinity Biosciences, DF13758). The following day, sections were rewarmed at room temperature for 30 min, then washed with phosphate‐buffered saline (PBS) three times, and incubated with secondary antibody (Alexa Fluor 594 and Alexa Fluor 488, Abcam, ab150080 and ab 150077) at 37°C for 1 h. Then, sections were washed with PBS three times, stained with DAPI for 15 min, and then washed with PBS. Finally, the brain sections were imaged using a fluorescence microscope (Nikon, Japan). The field of interest is the cerebral cortex around the injury.

### 2.8 Enzyme‐Linked Immunosorbent Assay (ELISA)

After the mice were euthanized, the brain tissue of the perihematomal area was removed and homogenized. ELISA kits (EK201BHS‐96 and EK218‐48, Multi Sciences) were used to detect the concentrations of IL‐1β and IL‐18, according to the manufacturer's instructions.

### 2.9 Western Blot

Proteins from brain tissue and cultured cells were extracted using radio immunoprecipitation assay (RIPA) buffer (Solarbio, R0020) supplemented with 1% phosphatase inhibitor at 4°C. Then, protein lysate was centrifuged for 20 min at 12,000 *g* at 4°C. After centrifuging, the supernatant protein solution was collected. After loading an equal amount of protein onto the gel, the electrophoresis procedure was initiated. The proteins were then transferred to PVDF membranes and blocked with QuickBlock Blocking Buffer for Western Blot (Beyotime, P0252, China) for 1 h at room temperature. After blocking, membranes were incubated with primary antibodies at 4°C overnight. The following primary antibodies were used: ASIC1a (1:1000, Proteintech, 27235‐1‐AP), GSDMD‐N (1:1000, Affinity Biosciences, DF13758), NLRP3 (1:1000, Proteintech, 30109‐1‐AP), ASC (1:5000, Proteintech, 10,500‐1‐AP), Cleaved‐Caspase 1 (1:1000, Zenbio, 341,030), NF‐κB p65 (1:1000, Proteintech, 10745‐1‐AP), Phospho‐NF‐κB p65 (1:2000, Proteintech, 80,379–2‐RR), β‐actin (1:1000, Beyotime Biotechnology, AF5003), GAPDH (1:50000, Proteintech, 60004‐1‐Ig). After washing three times with TBST, the membranes were incubated with secondary antibody at room temperature for 1 h and then washed a further three times. Finally, a Molecular Imager (Servicebio, China) was used to detect protein signals, which were quantified by Image J.

### 2.10 Cell Culture

HT‐22 neuronal cells and BV2 microglia cells were purchased from Procell Life Science & Technology (Wuhan, China). The cells were cultured in Dulbecco's modified Eagle's medium (DMEM) glucose containing 100 U/mL penicillin–streptomycin and 10% fetal bovine serum, and incubated in an incubator containing 5% CO_2_ at 37°C. BV2 cells were seedeed at 1 × 10^5^ cells per well in the upper chamber of Transwell plates, and mouse HT‐22 cells at 3 × 10^5^ cells per well in the lower chamber of Transwell plates. The plates were then incubated in a constant temperature incubator for 24 h. After the cells adhered, LPS at a concentration of 1 μg/mL was added to the medium of upper chamber microglia and co‐cultured for 24 h. The control group was treated with equal volumes of PBS or untreated medium. After the culture was completed, the neurons in the lower chamber were collected and subsequent detections were conducted.

### 2.11 Cell Viability Assay

The cell viability was determined using the CCK‐8 assay (Fdbio Science, China). Briefly, BV2 microglia cells were seeded on a 96‐well plate at a density of 1 × 10^4^ cells per well and treated with different concentrations of PcTx1 (5, 10, 20, 40 and 80 nM) for 24 h. Then, 10 μL/well of CCK‐8 solution was added to each well. After incubation for 2 h at 37°C, the optical density (OD) was measured using a microplate reader (SYNERGY H1 BioTek, USA) at 450 nm. Cell viability is represented by the OD value.

### 2.12 Quantitative Real‐Time PCR

Total RNA was isolated from peri‐contusional cortical tissues of TBI mice at 24 h post‐injury using the RNA extraction kit (Takra, 9767) according to the manufacturer's protocol. RNA was reverse‐transcribed into cDNA using the Hiscript II QRT Supermix for qPCR (Vazyme, R222‐01, China) according to the manufacturer's protocol. qPCR was immediately performed using the synthesized cDNA on an ABI 7500 Real‐Time PCR System (Applied Biosystems). Glyceraldehyde‐3‐phosphate dehydrogenase (GAPDH) served as the endogenous control. Relative gene expression was calculated using the 2^−ΔΔCt^ method. The sham group was used as reference. Primer sequences are provided in Table 1.

**TABLE 1:** Primers used for qRT‐PCR.

Name	Sequences (5′‐3′)
*Nlrp3‐F*	CTCTGTTCACTGGCTGCGGATG
*Nlrp3‐R*	TGGTCCTTTCCTCACGGTCTCC
*Asc‐F*	GCAACTGCGAGAAGGCTATGGG
*Asc‐R*	CTCATCTTGTCTTGGCTGGTGGTC
*Casp‐1‐F*	GCCGTGGAGAGAAACAAGGAGTG
*Casp‐1‐R*	CTATCAGCAGTGGGCATCTGTAGC
*Gapdh‐F*	AGGTCGGTGTGAACGGATTTG
*Gapdh‐R*	TGTAGACCATGTAGTTGAGGTGA

### 2.13 Statistical Analysis

All statistical analyses were performed using GraphPad Prism software (version 9.0). Data are presented as the mean ± SEM. For comparisons between two independent groups, an unpaired Student's *t*‐test was used. For comparisons among three or more groups, a one‐way or two‐way analysis of variance (ANOVA) was performed, followed by Tukey's honestly significant difference (HSD) post hoc test for multiple comparisons where ANOVA indicated significance. All experiments were conducted at least in triplicate. A *p*‐value < 0.05 was considered statistically significant.

## 3 Results

### 3.1 Temporal Patterns and Spatial Expressions of ASIC1a Following TBI

**FIGURE 2:**
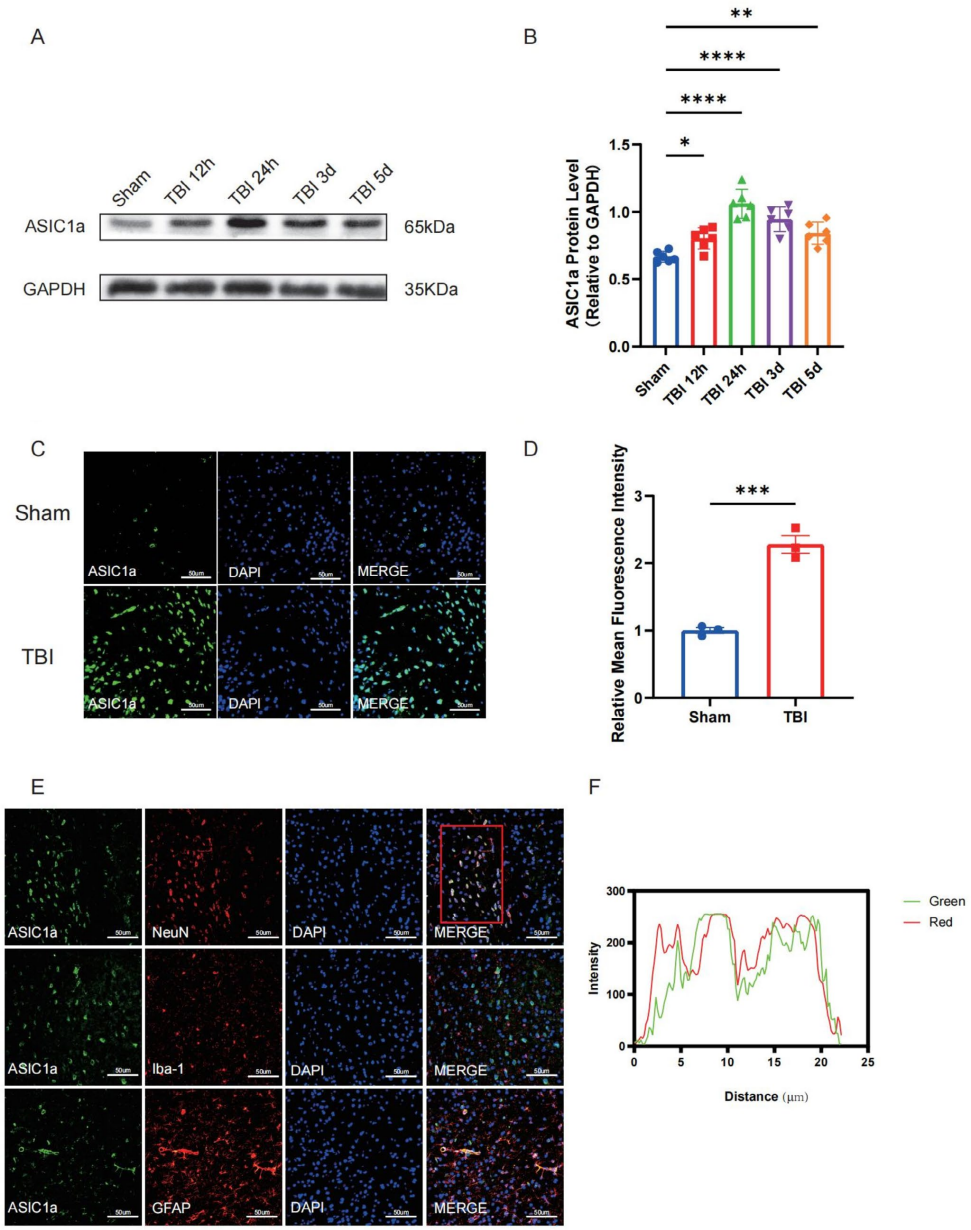
Temporal patterns and spatial expressions of ASIC1a following TBI. (A) Representative western blot band of temporal expression of ASIC1a following TBI. (B) Protein quantitative analysis of ASIC1a after TBI, *n* = 6 for each group. (C) Immunofluorescence staining images of ASIC1a at 24 h following TBI. (D) Quantitative analysis of the mean fluorescence intensity of ASIC1a at 24 h after TBI, *n* = 3 for each group. (E) Double immunofluorescence staining images of ASIC1a co‐located with microglia (Iba‐1), astrocytes (GFAP), and neurons (NeuN) at 24 h following TBI. (F) Quantitative analysis of co‐localization of ASIC1a and neurons. Data are expressed as the mean ± SEM. *p < 0.05, **p < 0.01, ***p < 0.001, ****p < 0.0001.

To investigate temporal ASIC1a expression patterns following traumatic brain injury, cerebral cortex samples were harvested at designated intervals (12 h, 24 h, 3 days, 5 days post‐TBI) for Western blot analysis. We found that, compared with the sham group, ASIC1a protein expression initiated at 12 h post‐TBI, peaking at 24 h (Figure 2A,B). Based on the protein expression results, immunofluorescence staining revealed significantly enhanced ASIC1a fluorescence intensity in the TBI group versus the sham group (Figure 2C,D). To explore the specific cellular localization of ASIC1a after TBI, dual immunofluorescence staining was used to detect the co‐localization analysis of ASIC1a with the neuronal marker NeuN, the microglial marker Iba‐1, and the astrocyte marker GFAP. We observed predominant ASIC1a localization within neurons of the peri‐contusional cortex (Figure 2E,F).

### 3.2 Inhibition of ASIC1a Attenuates Brain Edema and Ameliorates Neurological Deficits Following TBI

**FIGURE 3:**
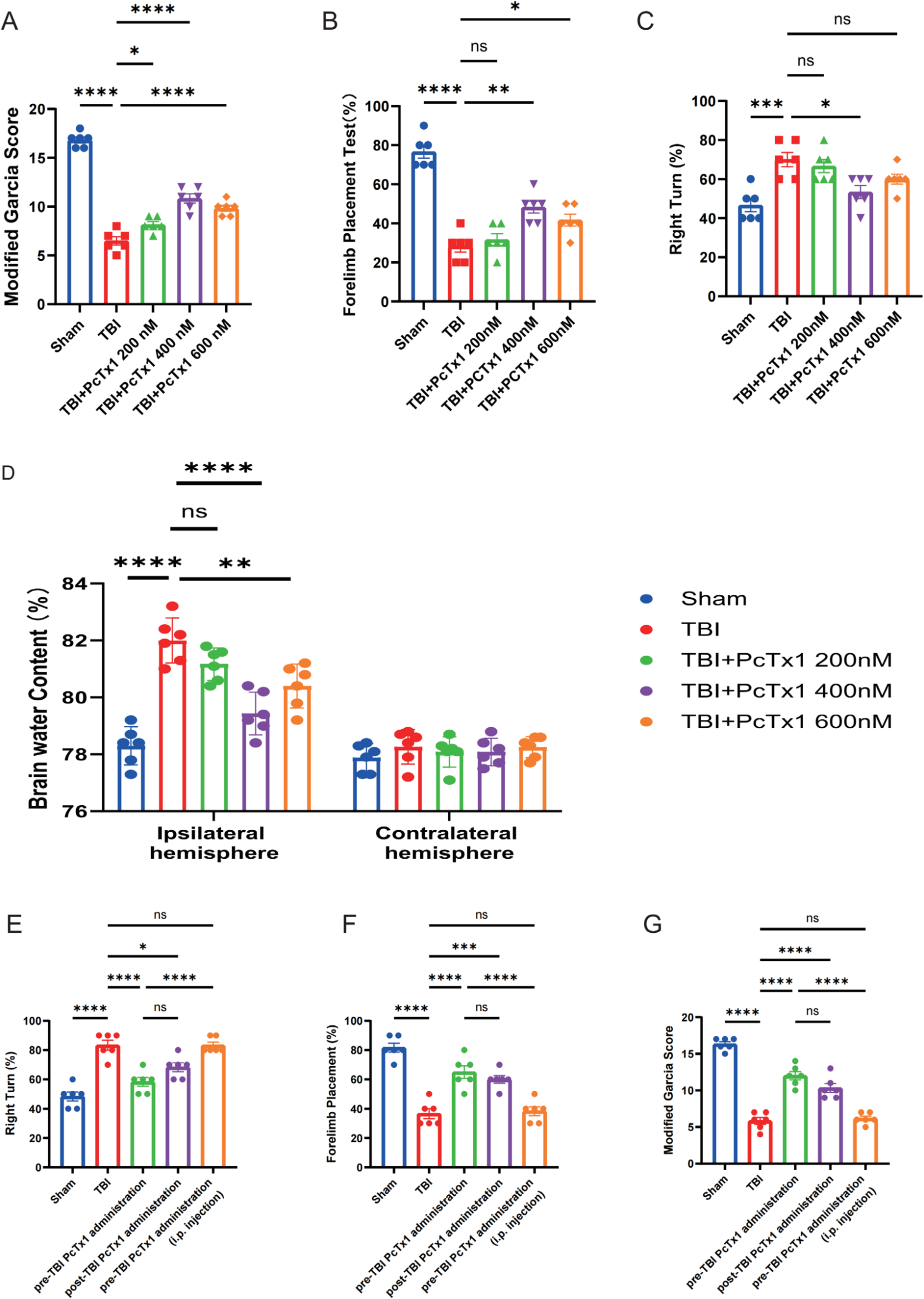
Inhibition of ASIC1a attenuates brain edema and ameliorates neurological deficits following TBI. (A) Modified garcia score. (B) Forelimb placement test. (C) Corner turn test. (D) Brain water content at 24 h following TBI. (E–G) Modified garcia score, forelimb placement test and corner turn test. *n* = 6 for each group. Data are expressed as the mean ± SEM. *p < 0.05, **p < 0.01, ***p < 0.001, ****p < 0.0001.

To investigate the therapeutic potential of ASIC1a inhibition, TBI mice received intranasal administration of the ASIC1a inhibitor PcTx1 at three concentrations (200, 400, 600 nM). The results of the modified Garcia score, forelimb placement test, and corner turn test indicated that TBI induced significant neurological impairment, compared with the sham group at 24 h. 200 and 600 nM PcTx1 administration did not attenuate the neurological deficits effectively, while the administration of 400 nM PcTx1 significantly improved neurological outcomes (Figure 3A–C). Meanwhile, the results of brain tissue water content suggested that the water content of the ipsilateral hemisphere significantly increased after 24 h in the TBI groups compared to the sham group, suggesting that TBI generated severe edema. However, treatment with 400 nM PcTx1 significantly reduced brain edema compared with treatment with 200 nM and 600 nM PcTx1 (Figure 3D). Therefore, a dose of 400 nM was selected for the following studies. To further investigate whether intranasal administration of PcTx1 after TBI also exerts neuroprotective effects, we administered 400 nM PcTx1 intranasally to mice 30 min post‐TBI and performed the same behavioral analyses at 24 h after TBI. Our results indicated that, compared with the TBI group, both the pre‐TBI PcTx1 administration group and the post‐TBI PcTx1 administration group exhibited significant improvements in neurological dysfunction, whereas no significant difference was observed between the pre‐TBI PcTx1 administration group and the post‐TBI PcTx1 administration group (Figure 3E,G). These observations suggest that both pre‐injury and post‐injury intranasal administration of PcTx1 may be associated with favorable effects on neurological function after TBI. Also, to investigate whether the neuroprotective effects of PcTx1 are mediated through central or peripheral pathways, we compared neurological function in mice receiving intranasal PcTx1 before TBI with those receiving intraperitoneal injection of PcTx1 before TBI. Results showed that, compared with the TBI group, mice administered intranasal PcTx1 tended to exhibit improved neurological function, whereas no obvious improvement in neurological function was observed in mice receiving intraperitoneal PcTx1. In addition, mice treated intranasally with PcTx1 appeared to display better neurological outcomes than those treated intraperitoneally (Figure 3E,G).

### 3.3 Inhibition of ASIC1a Alleviated Pyroptosis and Neuroinflammation Following TBI

**FIGURE 4:**
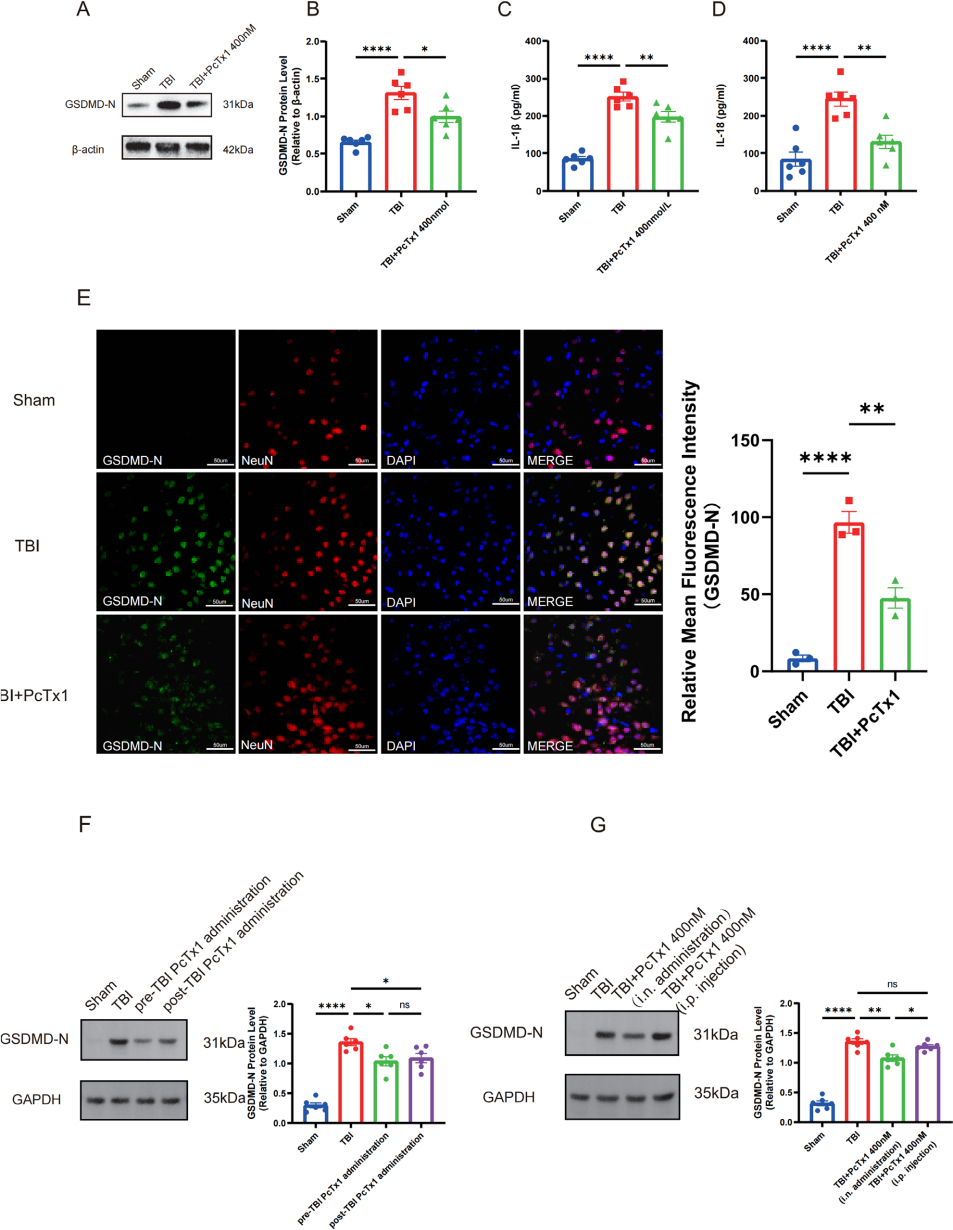
Inhibition of ASIC1a alleviated pyroptosis and neuroinflammation following TBI. (A) Representative western blots band of GSDMD‐N at 24 h following TBI. (B) Protein quantitative analysis of GSDMD‐N at 24 h following TBI, *n* = 6 for each group. (C) Concentrations of IL‐1β and IL‐18 (D) in the peri‐contusional cerebral cortex of mice according to ELISA, *n* = 6 for each group. (E) Double immunofluorescence staining images of GSDMD‐N co‐located with neurons (NeuN) and quantitative analysis of the mean fluorescence intensity of GSDMD‐N at 24 h following TBI, *n* = 3 for each group. (F, G) Representative western blots band and protein quantitative analysis of GSDMD‐N, *n* = 6 for each group. Data are expressed as the mean ± SEM. *p < 0.05, **p < 0.01, ****p < 0.0001.

To investigate the potential role of ASIC1a in promoting pyroptosis following TBI, we first examined the expression of the pyroptosis executioner protein GSDMD‐N in the peri‐contusional cortex at 24 h post‐TBI using Western blot analysis. Our results revealed a significant upregulation of GSDMD‐N expression after TBI, which was markedly attenuated by ASIC1a inhibition (Figure 4A,B). Consistent with these findings, ELISA analysis demonstrated substantial elevations in pyroptosis‐associated inflammatory cytokines IL‐1β and IL‐18 in the TBI group (Figure 4C,D). Notably, inhibition of ASIC1a significantly mitigated these TBI‐induced inflammatory responses. Furthermore, double immunofluorescence staining confirmed the occurrence of pyroptosis in cortical neurons post‐TBI, while ASIC1a inhibition reduced neuronal pyroptosis compared to TBI mice (Figure 4E). Similarly, we also examined the expression levels of GSDMD‐N following pre‐injury or post‐injury intranasal administration of PcTx1 in TBI mice. The result showed that, compared with the TBI group, both pre‐TBI and post‐TBI intranasal administration tended to reduce the expression of GSDMD‐N, and no significant difference was detected between the two treated groups (Figure 4F). To explore whether PcTx1 acts primarily through peripheral or central pathways, we also examined the expression of GSDMD‐N in TBI mice following intranasal or intraperitoneal administration of PcTx1. Results showed that, compared with the TBI group, GSDMD‐N expression tended to be decreased in mice receiving intranasal PcTx1, whereas no obvious changes in GSDMD‐N expression were observed in mice treated with intraperitoneal PcTx1 (Figure 4G). Collectively, these data provide compelling evidence that inhibition of ASIC1a alleviated pyroptosis and neuroinflammation after TBI.

### 3.4 Inhibition of ASIC1a Downregulates the NF‐κB/NLRP3 Axis and Attenuates Neuronal Pyroptosis Following TBI

**FIGURE 5:**
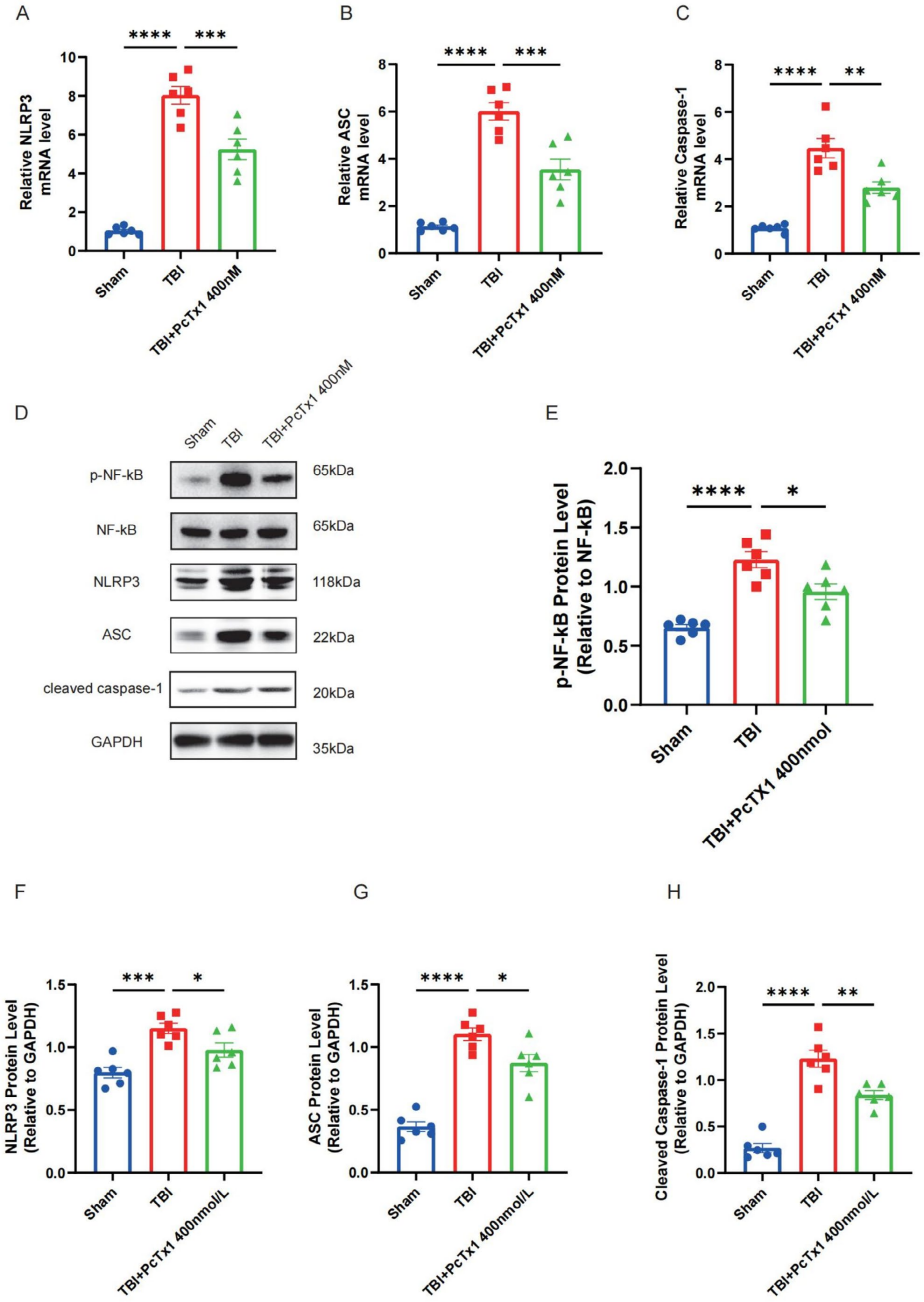
Inhibition of ASIC1a downregulates the NF‐κB/NLRP3 axis and attenuates neuronal pyroptosis following TBI. (A–C) Quantification of brain cortex *Nlrp3*, *Asc*, and *Casp‐1* mRNA level following TBI by qRT‐PCR, *n* = 6 for each group. (D–H) Representative western blots band and protein quantitative analysis of p‐NF‐κB, NLRP3, ASC, and Cleaved Caspase‐1, *n* = 6 for each group. Data are expressed as the mean ± SEM. *p < 0.05, **p < 0.01, ***p < 0.001, ****p < 0.0001.

To elucidate the molecular mechanism by which ASIC1a exacerbates pyroptosis following traumatic brain injury (TBI), we systematically investigated its potential involvement in the NF‐κB/NLRP3 inflammasome pathway. Quantitative PCR analysis revealed significant upregulation of *Nlrp3*, *Asc*, and *Casp‐1* in the TBI group compared to the sham group, while inhibition of ASIC1a substantially attenuated these transcriptional changes (Figure 5A–C). In addition, the protein expressions of phosphorylated NF‐κB (p‐NF‐κB), NLRP3, ASC, and Cleaved Caspase‐1 were increased in the TBI group compared to those in the sham group; however, the increments of these proteins were all markedly inhibited in TBI mice treated with PcTx1 (Figure 5D–H). Collectively, these results suggest that ASIC1a promotes neuronal pyroptosis following TBI, a process that is closely associated with the activation of the NF‐κB/NLRP3 signaling axis.

### 3.5 Inhibition of ASIC1a Attenuates Neuronal Pyroptosis in an In Vitro Model of Neuroinflammation

**FIGURE 6:**
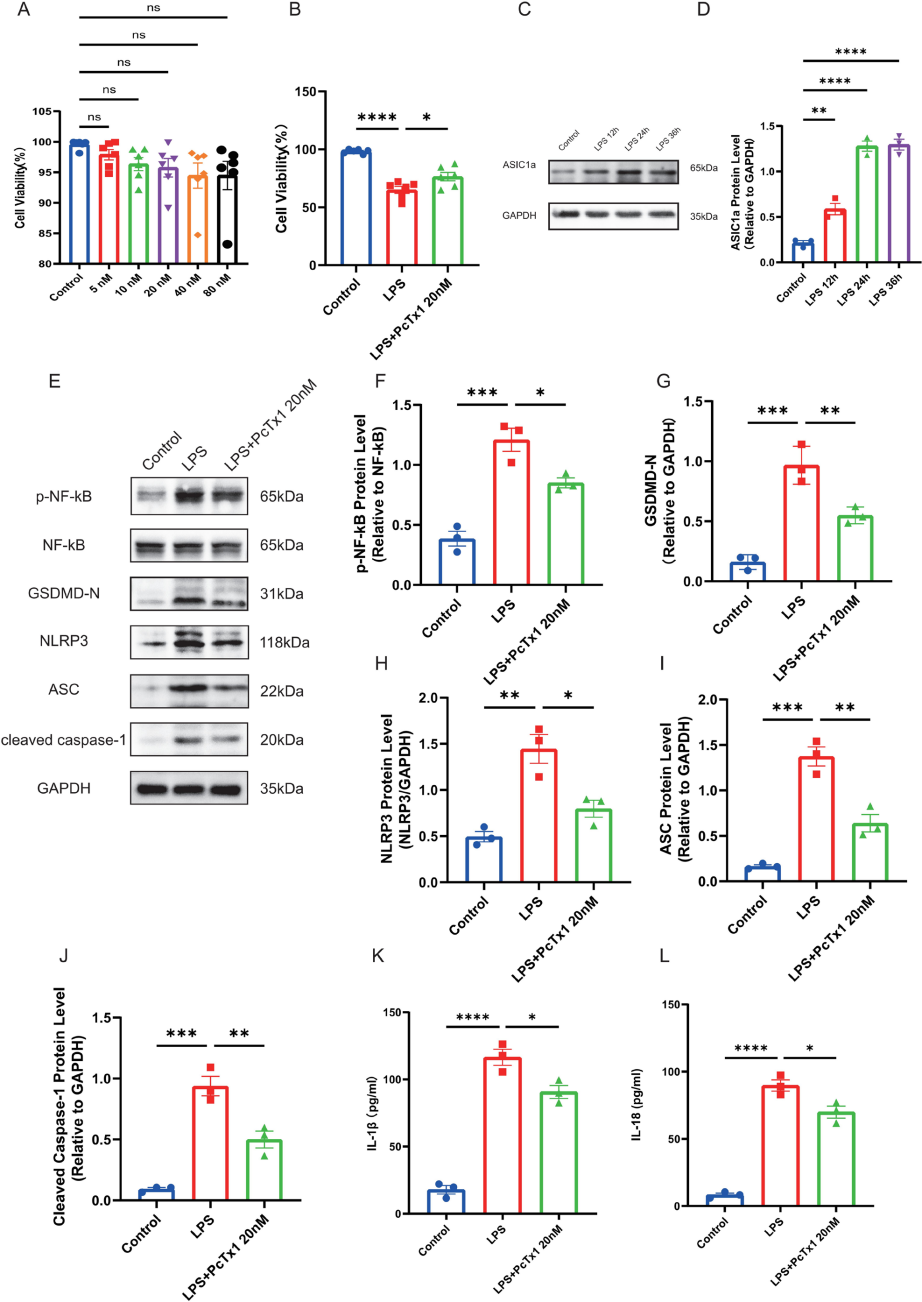
Inhibition of ASIC1a attenuates neuronal pyroptosis in an in vitro model of neuroinflammation. (A) The CCK‐8 assay was used to detect the toxic effects of different concentrations of PcTx1 on HT‐22 cells, *n* = 6 for each group. (B) The effects of LPS and PcTx1 on HT‐22 cell viability were detected by the CCK‐8 assay, *n* = 6 for each group. (C,D) Representative western blots band and protein quantitative analysis of ASIC1a expression in HT‐22 cells under different LPS stimulation times, *n* = 3 for each group. (E–J) Representative western blots band and protein quantitative analysis of p‐NF‐κB, GSDMD‐N, NLRP3, ASC, and Cleaved Caspase‐1, *n* = 3 for each group. (K, L) Concentrations of IL‐1β and IL‐18 according to ELISA, *n* = 3 for each group. Data are expressed as the mean ± SEM. *p < 0.05, **p < 0.01, ***p < 0.001, ****p < 0.0001.

To establish an in vitro neuroinflammatory model, we co‐cultured LPS‐stimulated BV2 microglia with HT‐22 neuronal cells and administered the selective ASIC1a inhibitor PcTx1. Initial CCK‐8 assays confirmed PcTx1 concentrations (5, 10, 20, 40, 80 nM) showed no cytotoxicity in HT‐22 cells (Figure 6A). Based on prior study [34], we selected 20 nM PcTx1 for subsequent experiments. LPS exposure (1 μg/mL for 24 h) significantly impaired HT‐22 viability when co‐cultured with BV2 microglia, which was partially rescued by ASIC1a inhibition (Figure 6B). Western blot analysis revealed dynamic ASIC1a upregulation in HT‐22 cells following LPS stimulation, with significant elevation at 12 h that plateaued by 24 h (Figure 6C,D). To verify whether ASIC1a also causes neuronal pyroptosis through the NF‐κB/NLRP3 pathway in vitro neuroinflammatory model, the expressions of GSDMD‐N, p‐NF‐κB, NLRP3, ASC and Cleaved Caspase‐1 in HT‐22 neuron cells after LPS stimulation for 24 h were detected by Western blot. We found that, compared with the control group, stimulated by LPS, GSDMD‐N, p‐NF‐κB, NLRP3, ASC, and Cleaved Caspase‐1 all increased significantly in HT‐22 cells stimulated by LPS, while all of which were reversed with inhibiting ASIC1a (Figure 6E–J). Meanwhile, ELISA result showed that the level of IL‐1β and IL‐18 increased significantly compared with the control group, while inhibition of ASIC1a reversed the increase (Figure 6K,L). Collectively, these in vitro results suggest that ASIC1a promotes neuronal pyroptosis, an effect that is associated with the activation of the NF‐κB/NLRP3 pathway.

### 3.6 Stimulation of ASIC1a Promotes NLRP3 Inflammasome Activation and Is Associated With NF‐κB Signaling in an In Vitro Neuroinflammatory Model

**FIGURE 7:**
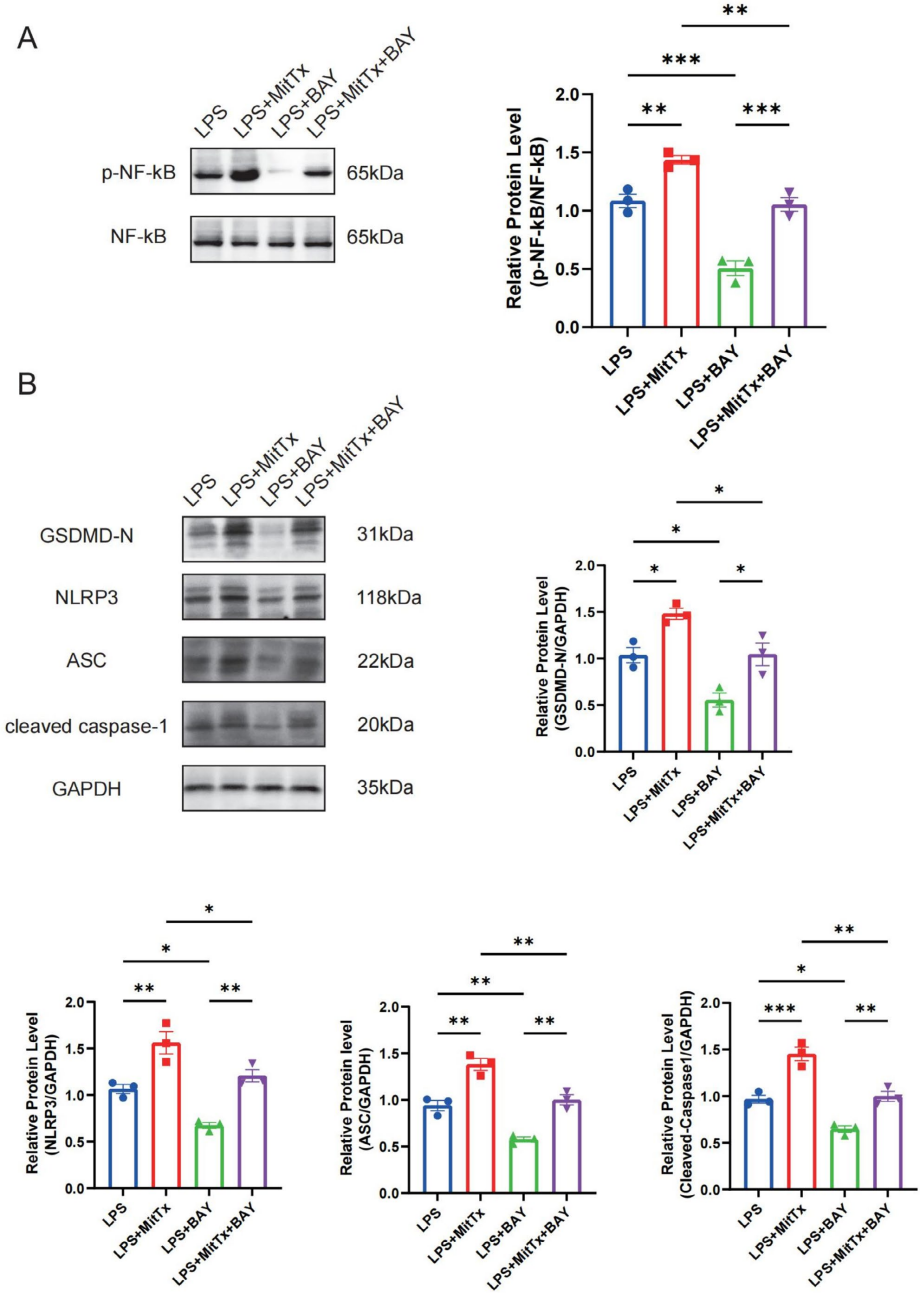
Stimulation of ASIC1a promotes NLRP3 inflammasome activation and is associated with NF‐κB signaling in an in vitro neuroinflammatory model (A) Representative western blots band and protein quantitative analysis of p‐NF‐κB, *n* = 3 for each group. (B) Representative western blots band and protein quantitative analysis of GSDMD‐N, NLRP3, ASC, Cleaved Caspase‐1, *n* = 3 for each group. Data are expressed as the mean ± SEM. *p < 0.05, **p < 0.01, ***p < 0.0001. In this specific mechanistic assay, the LPS‐alone group serves as the baseline control to evaluate the downstream modulatory effects of MitTx and BAY 11‐7082. For baseline validation relative to the untreated control, please refer to Figure 6.

Having established the successful LPS‐induced activation of pyroptosis in HT‐22 cells (as verified with untreated controls in Figure 6), we utilized the LPS‐alone group as the baseline to further explore the mechanistic interaction between ASIC1a and the NF‐κB/NLRP3 pathway. Specifically, an ASIC1a agonist MitTx and a NF‐κB inhibitor BAY 11‐7082 were administered prior to LPS treatment in HT‐22 cells, respectively. We found that phosphorylation of NF‐κB was significantly upregulated in the LPS + MitTx group compared with the LPS group, whereas BAY 11–7082 significantly reversed the MitTx‐induced upregulation of NF‐κB phosphorylation (Figure 7A). Meanwhile, compared to the LPS group, protein expression of GSDMD‐N, NLRP3, ASC, and Cleaved Caspase‐1 was significantly upregulated in the LPS + MitTx group, but significantly downregulated in the LPS + BAY 11‐7082 group. Furthermore, BAY 11‐7082 significantly reversed the MitTx‐induced upregulation of GSDMD‐N, NLRP3, ASC, and Cleaved Caspase‐1 protein expression (Figure 7B). Therefore, ASIC1a modulates the NLRP3 inflammasome, a process that is closely linked to the NF‐κB signaling pathway, thereby contributing to neuronal pyroptosis.

## 4 Discussion

**FIGURE 8:**
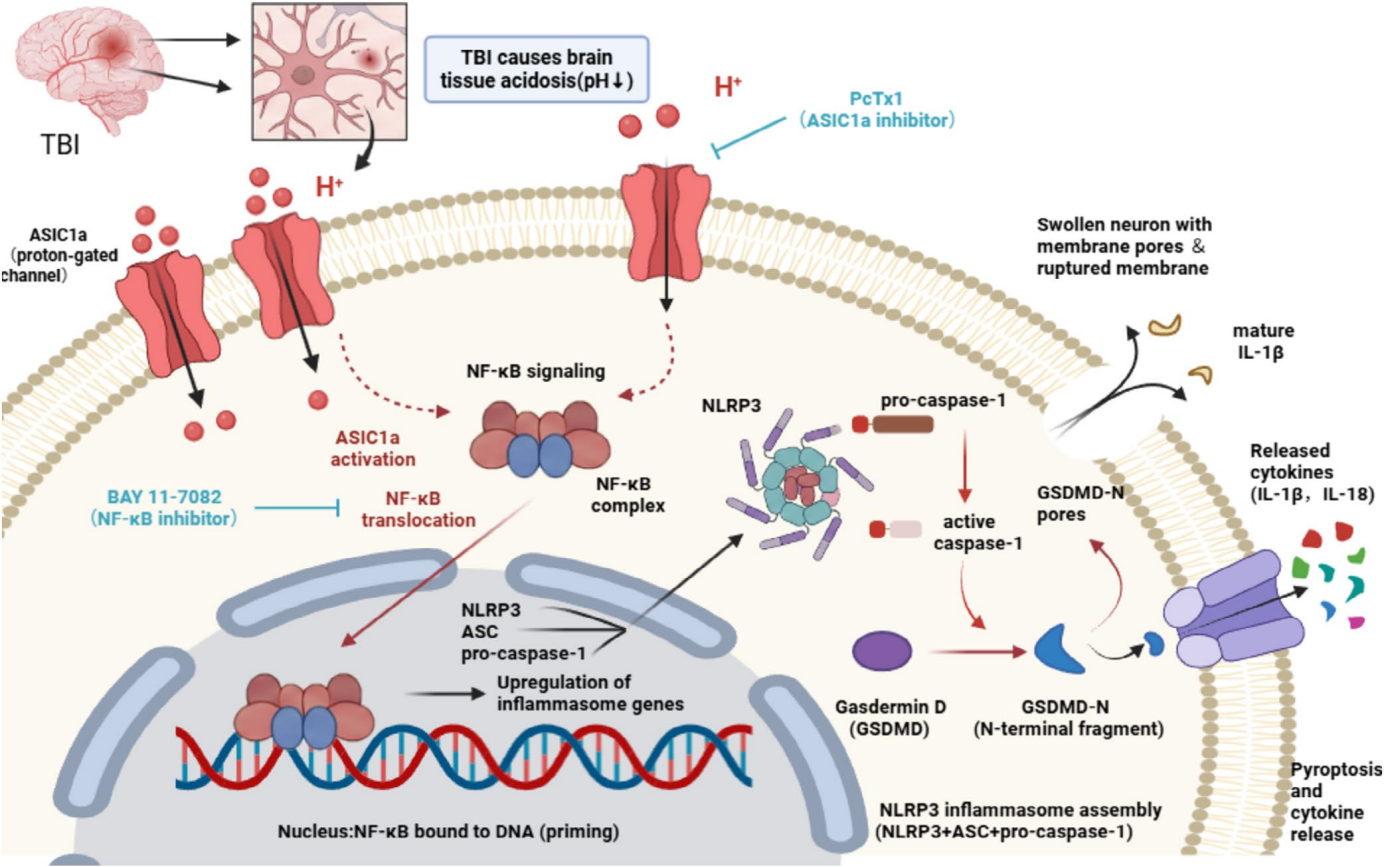
Proposed mechanism underlying ASIC1a‐modulated neuronal pyroptosis and neuroinflammation following TBI.

While the detrimental effects of ASIC1a have been well‐characterized in ischemic stroke, primarily via acidotoxicity, its specific role in the unique neuroinflammatory cascade following TBI remains poorly understood. Here, we provide novel evidence highlighting the important role of acid‐sensing ion channel 1a (ASIC1a) in neuroinflammatory responses following traumatic brain injury (TBI). Firstly, our findings revealed that ASIC1a protein expression was significantly upregulated post‐TBI, peaking at 24 h, with predominant neuronal localization. In addition, pharmacological inhibition of ASIC1a using PcTx1 markedly attenuated TBI‐induced neurological deficits and cerebral edema. Moreover, ASIC1a blockade effectively suppressed neuronal pyroptosis and was associated with reduced activation of the NF‐κB/NLRP3 signaling pathway. Collectively, these results suggest that ASIC1a inhibition mitigates neuroinflammation in TBI, a protective effect that is closely linked to the downregulation of the NF‐κB/NLRP3 pyroptotic axis (Figure 8).

Acidosis has been widely recognized as a critical contributor to the pathophysiology of secondary brain injury following TBI [35, 36, 37]. The extracellular pH begins to decline post‐TBI, with the magnitude of this decrease correlating with injury severity and poor clinical outcomes [38, 39, 40, 41]. Although acidosis can induce and aggravate neuronal damage [37], for example, through protein denaturation [42], induce cell swelling [43], or hinder mitochondrial energy metabolism [44], its exact damage mechanism is not yet fully understood. ASICs are fundamentally characterized by their high sensitivity to pH fluctuations, serving as critical molecular sensors for detecting subtle pH changes during synaptic activity or pathological acidosis in the brain [45, 46]. Among the six cloned mammalian subunits (ASIC1a, ASIC1b, ASIC2a, ASIC2b, ASIC3, and ASIC4), ASIC1 and ASIC2 represent the predominant subtypes in the central nervous system [47]. Most current studies have focused on ASIC channel subtype ASIC1a, which is most abundantly expressed in the central nervous system (CNS). Current research primarily focuses on ASIC1a—the most abundantly expressed ASIC subtype in the CNS. Functionally, ASIC1a operates as a pH‐dependent sodium/calcium‐permeable channel predominantly localized to postsynaptic membranes of central and peripheral neurons [48]. This channel facilitates excitatory neurotransmission, thereby mediating synaptic plasticity and memory formation under physiological conditions [49]. However, accumulating evidence highlights its significant involvement in various neuropathological conditions, particularly ischemic brain injury [50, 51]. Importantly, ASIC1a has been functionally implicated in acute neuronal damage following traumatic brain injury [52]. These findings collectively position ASIC1a as a promising therapeutic target for various neurological disorders characterized by pathological acidosis.

In this study, ASIC1a protein expression in the peri‐contusional cerebral cortex of mice exhibited a time‐dependent increase following traumatic brain injury (TBI), with upregulation initiating at 12 h post‐TBI, peaking at 24 h, and maintaining elevated levels despite a declining trend at 3 and 5 days. Immunofluorescence staining confirmed significantly enhanced ASIC1a mean fluorescence intensity in the TBI group compared to sham controls. Dual‐labeling immunofluorescence further demonstrated predominant co‐localization of ASIC1a with neuronal cells post‐TBI. In vitro, LPS stimulation of a co‐culture model comprising murine HT‐22 neuronal cells and BV2 microglial cells replicated this temporal pattern, showing marked ASIC1a upregulation in HT‐22 cells beginning at 12 h and stabilizing by 24 h. Collectively, these findings indicate that ASIC1a likely contributes to neuroinflammation after TBI.

PcTx1, a protein toxin that selectively binds to the subunit‐subunit interface of ASIC1a, inhibits channel activity by enhancing its apparent affinity for H^+^ ions [53]. In this study, different concentrations of PcTx1 were administered to TBI mice to observe their therapeutic effects on neurological deficits and cerebral edema in TBI mice. The results showed that 400 nM PcTx1 could effectively alleviate the neurological deficits and cerebral edema caused by TBI. This aligns with the neuroprotective effects demonstrated in Simon and Xiong's patent, wherein PcTx1 reduced infarct volume by 60% in cerebral ischemia models following targeted delivery, though intravenous administration failed to confer significant protection [34]. In vitro, PcTx1 (20 nM) attenuated LPS‐induced cytotoxicity in HT‐22 neurons via cell viability assays. Collectively, PcTx1 emerges as a potent ASIC1a inhibitor capable of mitigating TBI‐related neurological deficits, cerebral edema, and neuroinflammation.

Subsequently, this study investigated the mechanistic link between ASIC1a and neuronal pyroptosis. Pyroptosis represents an inflammatory form of programmed cell death characterized by the extracellular release of pro‐inflammatory cytokines. The pyroptosis executor GSDMD‐N forms cytotoxic pores in the plasma membrane, facilitating the release of inflammasome‐associated cytokines (IL‐1β and IL‐18) into the cytoplasm [54]. The NLRP3 inflammasome—comprising NLRP3, ASC, and pro‐caspase‐1—activates upon stimulation, leading to the cleavage of pro‐caspase‐1 into active caspase‐1 [55]. This protease then processes both pro‐IL‐1β/pro‐IL‐18 and gasdermin D (GSDMD) into its pore‐forming N‐terminal fragment (GSDMD‐N), executing pyroptotic cell death [56]. Notably, ASIC1a has been implicated in NLRP3‐mediated pyroptosis within intervertebral disc degeneration models [57]. Given that NF‐κB serves as a canonical activator of NLRP3 transcription and is triggered by acidosis [58], we hypothesized that ASIC1a exacerbates TBI‐induced neuronal pyroptosis in a manner associated with the NF‐κB/NLRP3 axis. Interestingly, we found that the mRNA levels of *Nlrp3*, *Asc*, and *Casp‐1* were upregulated following TBI compared to those in the sham group, while PcTx1‐mediated ASIC1a inhibition significantly reversed these increases. Additionally, the protein expression of GSDMD‐N, p‐NF‐κB, NLRP3, ASC, and Cleaved Caspase‐1 was also increased following TBI, while ASIC1a inhibition reversed this upregulation. Meanwhile, in in vitro experiments, LPS‐stimulated HT‐22 cells in co‐culture models recapitulated these increases. Moreover, the upregulation of NF‐κB/NLRP3 pathway‐related proteins induced by the ASIC1a agonist MitTx was significantly attenuated upon treatment with the NF‐κB inhibitor BAY 11‐7082 in HT‐22 cells. Thus, these findings suggest that ASIC1a promotes neuroinflammation post‐TBI, a process that is closely linked to the modulation of NF‐κB/NLRP3‐mediated neuronal pyroptosis. Blockade of ASIC1a ameliorates this pathogenic cascade, providing novel insight into the function and role of ASIC1a in regulating neuroinflammatory injury after TBI.

Several limitations of the present study should be acknowledged. First of all, our mechanistic findings rely primarily on pharmacological inhibition. While PcTx1 demonstrates high affinity for ASIC1a, the lack of genetic approaches (such as ASIC1a^−/−^ mice) prevents us from establishing definitive causality between ASIC1a and the NF‐κB/NLRP3 pyroptotic axis. Furthermore, the dissociation between histological outcomes and functional recovery at the 600 nM dose suggests a ceiling effect and potential off‐target toxicity. Comprehensive electrophysiological profiling is warranted to delineate the specificity and safety window of high‐dose PcTx1. Finally, the in vitro neuron–microglia co‐culture system represents a simplified model of neuroinflammation and does not fully capture the complex multicellular immune interactions—including astrocytic responses and leukocyte infiltration—or the regional heterogeneity present in vivo following TBI. Despite these limitations, our study provides valuable initial evidence that ASIC1a modulation holds promise as a therapeutic strategy for TBI.

## 5 Conclusion

In summary, our study provides novel evidence suggesting that ASIC1a contributes to neuroinflammation and neuronal pyroptosis following traumatic brain injury, a process that is closely associated with the NF‐κB/NLRP3 signaling pathway. However, the precise regulatory mechanisms and direct molecular interactions between ASIC1a and NF‐κB require further investigation. Collectively, these results suggest that pharmacological modulation of ASIC1a holds promise as a therapeutic strategy for mitigating secondary brain injury after TBI.

## Author Contributions

Conceptualization: Jiawei Liang, Peng Chen, and Shiqi Cheng. Methodology: Jiawei Liang, Peng Chen, and Shiqi Cheng. Software: Jiawei Liang, Yangyang Zhao, and Jia‐hong Cai. Formal analysis: Jiawei Liang. Investigation: Jiawei Liang, Pan Lei, Yulong Li. Resources: Yichen Zhang and Yong‐an Jiang. Data curation: Jiawei Liang and Peng Chen. Writing – original draft preparation: Jiawei Liang. Writing – review and editing: Yan Zhang and Shiqi Cheng. Visualization: Jiawei Liang and Peng Chen. Supervision: Yan Zhang and Shiqi Cheng. Project administration: Yan Zhang and Shiqi Cheng. Funding acquisition: Yan Zhang and Shiqi Cheng. All authors have read and agreed to the published version of the manuscript.

## Funding

This study was supported by grants from the National Natural Science Foundation of China (82260388, 82260378), the Natural Science Foundation of Jiangxi Province for Distinguished Young Scholars (20232ACB216009), the Natural Science Foundation of Jiangxi Provincial Department of Science and Technology—Key Project (20232ACB206019), the Key project of the Administration of Traditional Chinese Medicine of Jiangxi Province (2022Z017, SZYZD20233084), and the Jiangxi Province's “Double Thousand” High‐End Talent Project for Scientific and Technological Innovation (G3423).

## Ethics Statement

All animal procedures were performed in accordance with the Guidelines for Care and Use of Laboratory Animals of Nanchang University and approved by the Animal Ethics Committee (Approval No. NCULAE‐20221108001).

## Conflicts of Interest

The authors declare no conflicts of interest.

## Data Availability

The data that support the findings of this study are available on request from the corresponding author. The data are not publicly available due to privacy or ethical restrictions.
